# The “needle re-entry” technique for infrainguinal arterial calcified occlusive lesions

**DOI:** 10.1186/s42155-021-00274-y

**Published:** 2021-12-11

**Authors:** Takuya Haraguchi, Yoshifumi Kashima, Masanaga Tsujimoto, Tomohiko Watanabe, Hidemasa Shitan, Takuro Sugie, Daisuke Hachinohe, Umihiko Kaneko, Ken Kobayashi, Daitaro Kanno, Katsuhiko Sato, Tsutomu Fujita

**Affiliations:** Department of Cardiology and Head of Peripheral Artery Disease Center, Sapporo Heart Center, North 49, East 16, 8-1, Higashi ward, Sapporo, Hokkaido 007-0849 Japan

**Keywords:** Femoropopliteal artery disease, Endovascular intervention, Chronic total occlusions, Calcified plaque, Peripheral arterial disease, Stent graft, Re-entry

## Abstract

**Background:**

Vascular calcification is a predictor of poor clinical outcome during and after endovascular intervention. Guidewire crossing techniques and devices have been developed, but chronic total occlusions (CTOs) with severe calcification often prevent subintimal re-entry. We propose a novel guidewire crossing approach combined needle rendezvous with balloon snare technique, named the “needle re-entry” technique, for treatment of complex occlusive lesions.

**Main text:**

A 73-year-old female with severe claudication in her right calf with ankle brachial index of 0.62, and a computed tomography angiogram showed a long occlusion with diffuse calcification in superficial femoral artery. She was referred to our department to have peripheral interventions. Since the calcified vascular wall of the lesion prevented the successful re-entry, the “needle re-entry” was performed. First, a retrograde puncture of the SFA, distally to the occlusion, was performed and an 0.018-in. guidewire with a microcatheter was inserted to establish a retrograde fashion. Second, an antegrade 5.0-mm balloon was advanced into a subintimal plane and balloon dilation at 6 atm was maintained. Third, an 18-gauge needle was antegradely inserted from distal thigh to the dilated 5.0-mm balloon. After confirming a balloon rupture by the needle penetration, we continued to insert the needle to meet the retrograde guidewire tip. Then, a retrograde 0.014-in. guidewire was carefully advanced into the needle hole, named the “needle rendezvous” technique. After further guidewire advancement to accomplish a guidewire externalization, the needle was removed. Finally, since the guidewire was passing through the 5.0-mm ruptured balloon, the balloon was withdrawn, and the guidewire was caught with the balloon and successfully advanced into the antegrade subintimal space, named the “balloon snare” technique. After the guidewire was advanced into the antegrade guiding sheath and achieved a guidewire externalization, an endovascular stent graft and an interwoven stent were deployed to cover the lesion. After postballoon dilation, an angiography showed a satisfactory result without complications. No restenosis, reintervention, and limb loss have been observed for one year follow-up period after this technique.

**Conclusions:**

The “needle re-entry” technique is a useful guidewire crossing technique to revascularize femoropopliteal complex CTOs with severe calcification which prevent the achievement of guidewire crossing with the conventional procedures.

**Supplementary Information:**

The online version contains supplementary material available at 10.1186/s42155-021-00274-y.

## Background

Vascular calcification are major contributors to interventional revascularization failure in up to 25% for femoropopliteal chronic total occlusions (CTOs) (Scheinert et al., 2001). Severe calcified plaques negatively affect postprocedural minimal lumen area and patency (Fujihara et al., 2019). Calcification is thought to be a predictor of poor clinical outcome after procedure.

Guidewire crossing techniques have been developed, but CTOs, especially with severe calcification, are still challenging lesions in peripheral intervention. Re-entry devices are often used for these lesions (Bausback et al., 2011), but the use of these device does not guarantee the technical success in all cases. We propose a novel guidewire re-entry approach by combination of needle rendezvous (Haraguchi et al., 2021) and balloon snare technique, named the “needle re-entry (NRE)” technique for complex calcified CTOs with impassable passage by conventional interventional techniques and devices.

## Main text

**Fig. 1:**
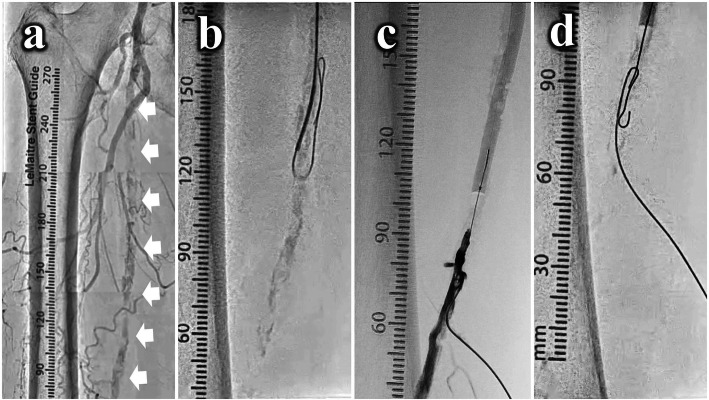
Angiography and the conventional bi-directional approach. **A**. Control angiography showed the overall lesion (white arrows). **B**. A 0.035-in. guidewire was antegradely advanced into the subintimal plane to perform percutaneous intentional extraluminal recanalization (PIER) technique, but antegrade re-entry wiring failed. **C**. The distal SFA was punctured with 20-gauge needle, and 0.018-in. guidewire with a microcatheter was inserted to establish a retrograde fashion, and the distal lumen was revealed by tip injection through the retrograde microcatheter. **D**. The retrograde wire could not be advanced into the occlusion due to the severely calcified cap

A 73-year-old female with diabetes mellitus was presented with severe claudication, Rutherford classification 3, in her right calf. Her right ankle brachial index was 0.62 and a computed tomography angiogram (CTA) showed a long CTO with diffuse calcification in her right superficial femoral artery (SFA). Since the patient was obese and had a short right common femoral artery (CFA) that was not suitable for an ipsilateral approach, a 7-Fr guiding sheath (Destination®^□^, Terumo Co., Japan) was percutaneously canulated to establish a crossover fashion from the left CFA. Angiography demonstrated a diffuse calcified CTO in right SFA (Fig. 1a). We intra-arterially injected 5000-IU of unfractionated heparin from the guiding sheath. The antegrade wiring was attempted with several hard guidewires supported with a microcatheter. However, the proximal calcified orifice prevented the guidewire penetration to enter the intraplaque. Therefore, we advanced the 0.035-in. guidewire with looped wire technique into the subintimal plane to attempt percutaneous intentional extraluminal recanalization (PIER) technique (Reekers et al., 1994) (Fig. 1b), but antegrade re-entry failed. The Outback system (Cardinal Health Inc., USA) was used to attempt re-entry, however, its needle could not penetrate the calcified wall of the distal lumen.

**Fig. 2:**
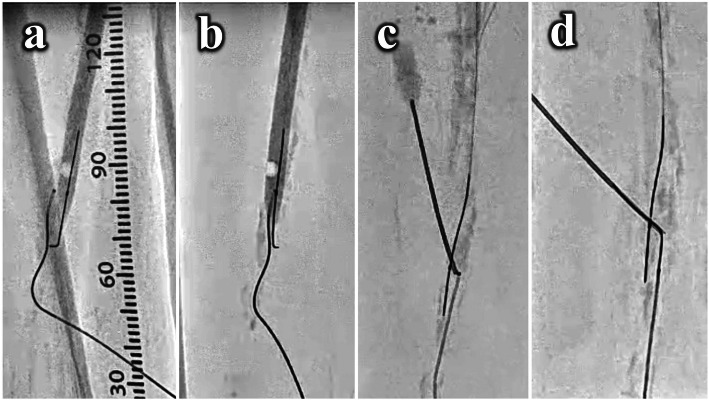
The process of the “needle rendezvous” of the “needle re-entry” technique. **A**. The retrograde microcatheter in the distal lumen below the dilated antegrade 5.0-mm balloon in the subintimal plane was confirmed in ipsilateral view. **B**. These devices were overlapped in contralateral view. **C**. An 18-gauge needle was inserted from the distal thigh to the dilated balloon. After confirming a balloon rupture by the needle penetration, the needle was continued to advance to meet the retrograde guidewire tip. **D**. A retrograde 0.014-in. guidewire was carefully manipulated and successfully advanced into the needle hole

**Fig. 3:**
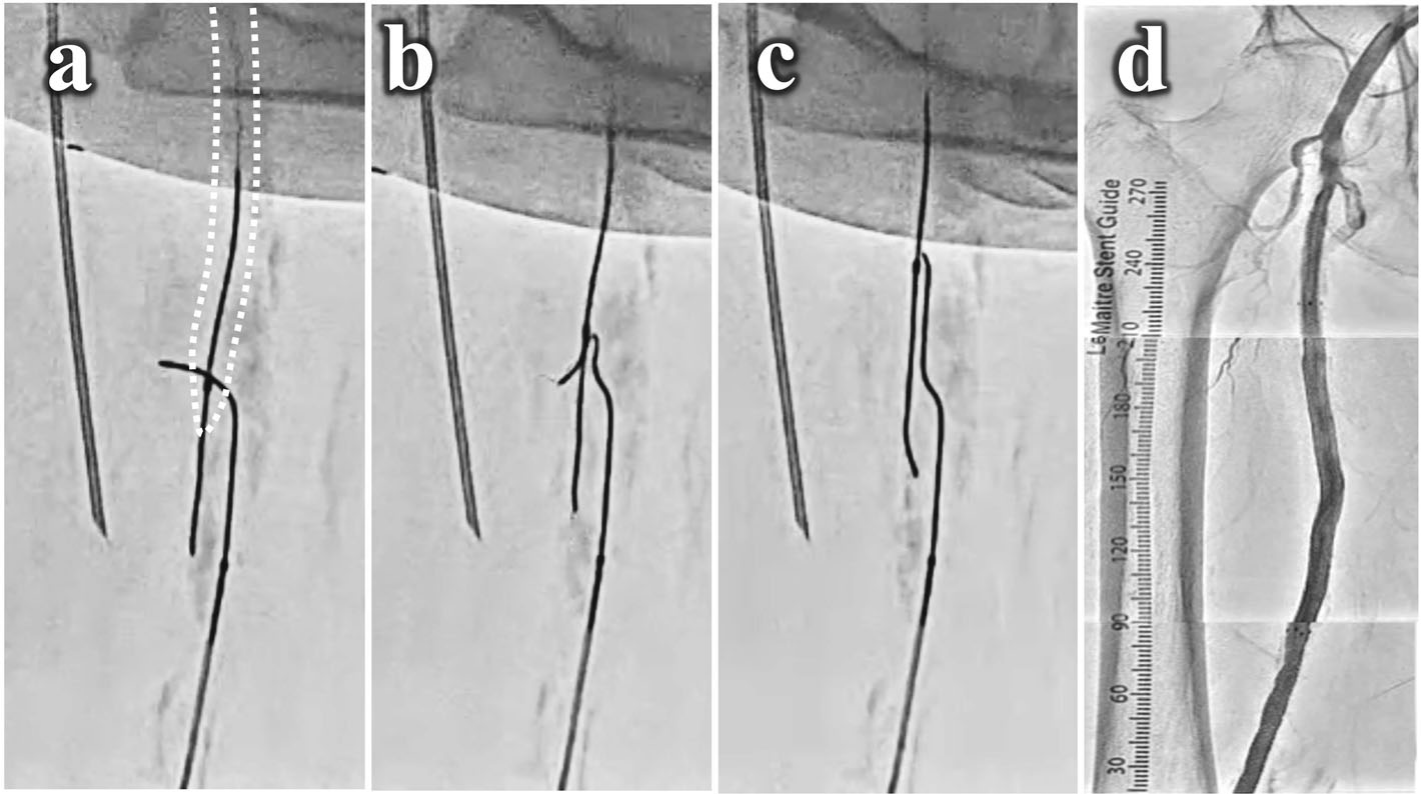
The process of the “balloon snare” of the “needle re-entry” technique. **A** to **C**. Since the retrograde guidewire passed into the 5.0-mm balloon (white dotted line), when the balloon was withdrawn, and the guidewire was caught with the balloon and successfully advanced into the antegrade subintimal space. **D**. After the “pave-and-crack” technique was performed, a 6.0 × 250-mm stent graft combined with a 6.5 × 150-mm interwoven stent were implanted, final angiography showed a satisfactory result without complications

Therefore, the antegrade strategy was changed to the retrograde one. We punctured the distal SFA with 20-gauge needle with fluoroscopic guidance and inserted 0.018-in. guidewire with a microcatheter to establish a retrograde fashion (Fig. 1c). The distal calcified cap obstructed the penetration of retrograde wiring even with the hard guidewire and looped wire technique (Fig. 1d). Thus, we performed the NRE technique which needle rendezvous technique is combined with balloon snare technique. First, a 5.0 × 120-mm noncompliant balloon (JADE®^□^, OrbusNeich, China) was antegradely advanced into the subintimal space and balloon dilatation at 6 atm was maintained. The position of retrograde microcatheter below the dilated antegrade balloon was confirmed in ipsilateral view (Fig. 2a), and these devices were overlapped in contralateral view (Fig. 2b). Second, we antegradely inserted an 18-gauge needle from the distal thigh to the dilated 5.0-mm balloon. After a balloon rupture by the needle penetration was confirmed, we continued to insert the needle to pass through the balloon and to meet the retrograde guidewire tip while confirming the correct direction and depth between the tips of the guidewire and the needle in contralateral and ipsilateral views (Fig. 2c). Then, a retrograde 0.014-in. guidewire was carefully manipulated and successfully advanced into the needle hole (Fig. 2d). This technique is named the “needle rendezvous” technique. Third, the needle was removed after further advancing the guidewire into the needle hole until the soft part of the guidewire, the radiopaque part, not the shaft, passed through the balloon (Fig. 3a). As the balloon was withdrawn, the guidewire was caught with the ruptured balloon material and pulled along together into the subintimal space (Fig. 3b). Finally, the balloon was pulled out, the guidewire came out of the balloon where the needle punctured, and eventually the guidewire remained in the antegrade subintimal space (Fig. 3c). This is the “balloon snare “technique. After the guidewire was advanced into the antegrade guiding sheath and accomplished a guidewire externalization, we performed the “pave-and-crack” technique (Dias-Neto et al., 2018) with implanting a 6.0 × 250-mm stent graft (Viabahn®^□^, W.L. Gore & Associated, Inc., USA) combined with a 6.5 × 150-mm interwoven stent (Supera®^□^, Abbott Vascular, USA). After the postballoon dilation with a 7.0 × 150-mm noncompliant balloon (SHIDEN HP®^□^, Kaneka Co., Japan) at highest pressure, an angiography and a final intravascular ultrasound showed a satisfactory result without complications (Fig. 3d). The patient’s symptom improved and ABI normalized after the procedure. Furthermore, restenosis has not been detected with ABI and a duplex scan for one year follow-up period.

## Discussion

This is the introduction to the NRE technique for guidewire re-entry to recanalize femoropopliteal complex CTOs with severe calcification, and the process of this technique is shown in the supplementary material (Supplemental movie 1). Several guidewire crossing procedures, such as PIER technique, subintimal arterial flossing with antegrade-retrograde intervention (SAFARI) technique, controlled antegrade and retrograde tracking and dissection (CART), and reverse-CART (Surmely et al., 2006) improve the technical success of subintimal crossing. Moreover, the Outback system, one of the re-entry devices, was used for antegradely crossing the CTOs with a technical success rate of 90.7% (Bausback et al., 2011). Also, the severely calcified occlusion in this case was too hard to be crossed with the conventional techniques and the Outback system. Our NRE technique using an 18-gauge needle penetrated the calcification between the distal lumen and antegrade subintimal space, eventually leading to successful re-entry. This technique may help to further increase technical success rate in the similar cases.

In terms of limitations, first, the NRE can be achieved in the infrainguinal arteries, but not in the suprainguinal arteries. Second, the NRE cannot be used if an antegrade balloon cannot be advanced into a subintimal plane. Third, patient with large diameter thigh could not be proper candidates with respect for this particular technique. Forth, if antegrade guidewire does not presents in a subintimal space in the front side of the vessel, our NRE technique will not be applied. While, if antegrade guidewire exists in a subintima in the back side of the vessel, we recommend performing the “poorman’s outback (POB)” (Urasawa, 2013). The POB is as follows. An 18-gauge needle is retrogradely inserted from a distal thigh and advanced through a distal lumen to a tip of antegrade wire in an antegrade subintimal plane. After confirming that the needle touches the guidewire tip, we advance the guidewire into the needle hole and remove the needle. Then, the guidewire is controlled to advance into the distal lumen to achieve the re-entry. The representative case treated with POB is in the supplemental material (Supplemental movie 2). Finally, there is a learning curve involved in puncturing through an expanded balloon into a subintimal space to reach a guidewire tip in a distal lumen and in the manipulation of the guidewire to advance into the needle hole.

## Conclusions

The NRE technique, which combined the needle rendezvous technique with the balloon snare technique, is a useful re-entry method to revascularize femoropopliteal complex CTOs with severe calcification which prevent the achievement of guidewire crossing. We should accumulate more cases to evaluate the efficacy of recanalization for calcified femoropopliteal occlusions.

## Supplementary Information

**Additional file 1.** Supplementary movie 1 The process of the “needle re-entry” technique.

**Additional file 2.** Supplemental movie 2 The process of the “poor man’s outback” technique.

## Data Availability

The datasets used and/or analyzed during the current study are available from the corresponding author on reasonable request.

## Abbreviations

CTO: Chronic total occlusion
NRE: Needle re-entry
CTA: Computed tomography angiogram
SFA: Superficial femoral artery
CFA: Common femoral artery
PIER: Percutaneous intentional extraluminal recanalization
SAFARI: Subintimal arterial flossing with antegrade-retrograde intervention
CART: Controlled antegrade and retrograde tracking and dissection
POB: Poorman’s outback
